# HIV-1 virion lysis following centrifugation improves the sensitivity of the Fourth-Generation HIV Ag/Ab combo assay

**DOI:** 10.1186/s13104-024-06810-y

**Published:** 2024-06-04

**Authors:** Allan Wandera, Kenneth Ssekatawa, Charles Drago Kato, Eliah Kwizera, Pastori Mujinya, Robert Siida

**Affiliations:** 1 Department of Biochemistry, Faculty of Biomedical Sciences, Kampala International University, Western-Campus, P.O Box 71, Bushenyi, Uganda; 2 Department of Science, Technical and Vocational Education, Makerere University, P. O. Box 7062, Kampala, Uganda; 3 Africa Center Excellence in Materials Product Development and Nanotechnology (MAPRONANO ACE), Makerere University, P. O. Box 7062, Kampala, Uganda; 4 Department of Biotechnical and Diagnostic Sciences, Makerere University, P.O Box 7062, Kampala, Uganda

**Keywords:** Centrifugation, HIV-1 virion lysis, Sensitivity, Fourth-generation combo assay

## Abstract

**Objective:**

Fourth-generation HIV Ag/Ab Combo assay is used for HIV screening of blood for transfusion in developing countries, however, the sensitivity of the assay is questionable during the acute phase of HIV infection. Thus, the study aimed to determine the effect of combining centrifugation with HIV-1 virion lysis on the sensitivity of the fourth-generation HIV Ag/Ab combo assay.

**Results:**

When the 50 HIV-1 antibody-negative samples were run on the fourth-generation HIV Ag/Ab combo assay, 8 (16%) were positive following centrifugation, 13 (26%) were positive following lysis while 25 (50%) were positive after combining centrifugation with HIV-1 virion lysis.

## Background

The Human Immunodeficiency Virus (HIV) belongs to the lentivirinae subfamily of retroviruses that cause Acquired Immunodeficiency Syndrome (AIDS) [1]. A report by the United Nations Program on HIV/AIDS estimated that since the start of the epidemic, a total of 77.3 million people have since been infected with HIV/AIDS, and of these, about 35.4 million people have died of AIDS-related illnesses [2].

In order to make sure that blood for transfusion is free from HIV, the 4th -generation HIV Ag/Ab combo test was recommended by WHO for use in most countries [3]. However, several studies have questioned the sensitivity of this approach [4]. This is because the 4th -generation assay is insensitive within the first three weeks following infection until the viral load is about 30,734 copies/ml when the p24 antigens are sufficient to be detected by the assay [5]. This therefore implies that using the 4th -generation HIV combo assay to screen blood for transfusion would mean that some samples whose donors were in the acute stage of HIV infection and whose viral loads are below 30,734 RNA copies/ml will not be detected and passed as safe for transfusion which puts the blood recipients at a very high risk of contracting the virus [4].

Previous recommendations suggested that combining centrifugation of blood plasma with HIV virion lysis could be a more cost-effective technique to improve the sensitivity of the assay. Because it requires less laborious sample manipulation procedures, it makes it applicable in the resource-limited settings in the Sub- Saharan Africa. This study therefore determined the effect of combining plasma centrifugation with HIV virion lysis on the sensitivity of 4th generation Architect Ag/Ab combo assay in the detection of acute HIV infection.

## Main text

### Methods and materials

#### Study site and sample size determination

The study was carried out at Central Public Health Laboratories (CPHL), Kampala, and the National Blood Bank Laboratory, Nakasero Kampala. The Yamane sampling technique was used to determine the sample size where a total of 84 Samples were used in the study [6]. Samples with clots or measuring less than 2 milliliters were excluded from the study.

### Sample retrieval

Plasma samples were retrieved from the refrigerators where they were kept at -80^o^ C and left to stand at room temperature for 30 min to thaw. Each sample was divided into four aliquots of 500 µl. Three of the four aliquots were used in the determination of each of the study-specific objectives.

### Experimental control samples

Out of the four 500 µl aliquots of plasma samples, one aliquot was not subjected to any treatment but was run with the fourth-generation Architect Ag/Ab combo assay to serve as the control. The study also included 25 PCR-negative (HIV negative) samples that were also subjected to the three procedures of centrifugation alone, lysis alone, and a combination of both centrifugation and lysis for determination of specificity in the assay improvement. This assay generates numerical results known as a signal–to–cut–off ratio (S/CO), which measures the strength of the detected signal in comparison to a predefined cutoff value. Reactivity was defined as a sample with a S/CO ratio greater than or equal to 1, which indicated the presence of HIV- p24 antigens above the cutoff level. In contrast, a S/CO ratio less than 1 denoted non-reactive, implying that insufficient HIV-p24 antigens were present to surpass the cutoff threshold. The other three aliquots were each subjected to either centrifugation, lysis, or both centrifugation and lysis before the p24 Ag testing.

### Sample centrifugation

This was done following the procedure described by [7]. Briefly, 500 µl of the aliquoted samples were centrifuged at 23,000 g at 4^o^C for 60 min. The supernatant was discarded and the pellet was re-suspended in 60 µl of Rosewell Park Memorial Institute media (RPMI) 1640 (Gibco), vortexed for 1 min, and 100 µl of this suspension was then run by the Fourth–Generation Architect Ag/Ab combo assay.

### Sample lysis

Sample lysis was done as described by [8] with some modifications. Briefly, 500 µl of the aliquoted sample was mixed with 250 µl of the lysis buffer containing [30mM Tris/HCl pH 7.2, 450mM NaCl, 1.5% Triton X-100, 1.5% deoxycholic acid, 0.3% sodium dodecyl sulfate, and 10mM EDTA] and left for 10 min at room temperature. The resultant suspension was then run through the fourth-generation Architect Ag/Ab combo assay.

### Combining centrifugation with lysis

In order to achieve this, 500 µl of the plasma sample aliquot was centrifuged as discussed previously. The resultant pellet was re-suspended in 200 µl of the lysis buffer and vortexed for 1 min, then run with the Fourth-Generation Architect Ag/Ab combo assay.

### Statistical analysis

The results obtained were recorded and analysis was done using Graph Prism version 7 software. The paired samples t-test was used to determine the significance of the observed differences. The p-value < 0.05 was considered statistically significant.

## Results

### Effect of centrifugation on detection of HIV-1 p24 antigens using the fourth-generation Architect Ag/Ab combo assay

Of the 50 PCR-positive HIV-1 antibody-negative samples that were centrifuged and run with the fourth-generation Architect Ag/Ab combo assay, 8 (16%) were positive (had an S/CO ratio of ≥ 1) (Table 1) with a minimum detection limit of 9,130 viral RNA copies/ml at an S/CO ratio of 1.128 (Table 2). When the paired t-test was performed, it was found that there was a statistically significant difference (*p* = 0.0058) in the detection efficiency of HIV-1 p24 antigens between the test and control samples at 95% Confidence interval. The sensitivity and specificity of this treatment were 16% and 100% respectively at viral loads of less than 30,734 HIV-1 RNA copies/ml. (Table 3).

**Table 1:** Detection of HIV-1 p24 antigen in plasma of centrifuged, lysed, and both centrifuged and lysed samples from PCR positive and PCR Negative Samples

	**Treatment/test**	**Number of samples**		**Percentage of PCR-positive (%)**
		**PCR positive**	**PCR negative**	
Detection of p24 antigen (Positives/total)	Centrifuged samples	8/50	0/25	16
	Lysed samples	16/50	0/25	26
	Centrifuged and lysed samples	25/50	0/25	50

**Table 2:** Numbers, percentages, minimum S/CO ratios, and corresponding minimum viral loads detected for various sample treatment methods

Treatment	Samples that tested positive	Percentages of samples that tested positive	Minimum S/CO ratio	Minimum viral load detected (RNA copies/ml)	*p*-value
**Control samples**	2	4%	1.161	28,700	
**Centrifugation**	8	16%	1.128	9,130	0.0058
**Lysis**	13	26%	1.212	5,416	0.0006
**Centrifugation and lysis**	25	50%	1.412	1,565	< 0.0001

**Table 3:** Sensitivities and specificities of the different sample treatment techniques at viral loads below 30,734 RNA copies/ml

	Centrifugation	Lysis	Combination of lysis and Centrifugation
**Sensitivity**	16%	26%	50%
**Specificity**	100%	100%	100%

### Effect of lysis on detection of HIV-1 p24 antigens using the 4th Generation Architect Ag/Ab combo assay

When the samples were lysed and run through the fourth-generation Architect Ag/Ab combo assay, 13 (26%) were positive (had an S/CO ratio of ≥ 1) (Table 1) with a minimum detection of 5,416 viral RNA copies/ml at an S/CO ratio of 1.212 (Table 2). The sample paired t-test showed that there was a statistically significant difference (*p* = 0.0006) in the detection efficiency of HIV-1 p24 antigens between the test and control samples at 95% Confidence Interval. The sensitivity and specificity of this treatment were 26% and 100% respectively at viral loads of less than 30,734 HIV RNA copies/ml. (Table 3).

### Effect of combining Centrifugation with HIV-1 Lysis on detection of HIV-1 p24 antigens using the fourth-generation Architect Ag/Ab combo assay

When the samples were centrifuged, lysed, and ran through the Architect Ag/Ab combo assay, 25 (50%) of the samples were positive (had a S/CO ratio of ≥ 1) with a minimum detection limit of 1,565 viral RNA copies/ml at a S/CO ratio of 1.412 with a statistically significant difference, *p* = 0.0001 at 95% confidence interval (Tables 1 and 2). The sensitivity and specificity of this treatment were 50% and 100% respectively at viral loads of less than 30,734 HIV RNA copies/ml (Table 3). When the 25 PCR-negative samples were run through all the above treatments, they all tested negative with the fourth-generation Architect Ag/Ab combo assay (Table 1). When the aliquots that were not subjected to any treatment were run through the Architect Ag/Ab combo assay, 2 (4%) of the samples were positive for p24 antigens with a **Minimum S/CO ratio of** 1.161 and a minimum detection limit of 28,700 RNA copies/ml (Table 2).

## Discussion

This study presents a modified technique to improve the detection of HIV-1 p24 antigens in the plasma of infected individuals during acute HIV-1 infection stages. The results show an improvement in the detection of p24 antigens in the plasma of infected individuals during acute HIV-1 infection stages when viral loads are still low in circulation. The sensitivity of 16% after centrifugation is less than the 75% reported by Farías et al., but this could be due to the small sample size used in the study. The paired-sample t-test showed a significant difference between the treatment and control samples, with sensitivity and specificity of 16% and 100% respectively at viral loads of less than 35,000 HIV RNA copies/ml. Sample lysis alone had a considerable improvement in the detection of p24 antigens in HIV-1 PCR-positive samples and EIA-negative samples. The relationship between p24 antigen detection and sample lysis is consistent with previous studies, showing a considerable improvement in the recovery of HIV-1 p24 antigens. The virus lysis buffer used in this study is very efficient and significantly improves the recovery of p24 antigens in the plasma of infected patients.

The combination of sample centrifugation and HIV-1 virion lysis significantly improved the detection of HIV-1 p24 antigens in samples centrifuged and lysed. The detection limit after sample lysis was 5,416 RNA copies/ml at a S/CO ratio of 1.212 with 13 positive samples, while after centrifugation, it was 9,130 RNA copies/ml at a S/CO ratio of 1.128 with 8 positive samples. When combined, the detection limit was 1,565 Viral RNA copies/ml at an S/CO ratio of 1.412 with 25 positive samples. This improvement was significantly better than other findings, which found the minimum detection limit of the Architect Ag/Ab combo assay to be 35,000 HIV RNA copies/ml at an S/CO ratio of 0.2 [9]. While this study focuses on the physical manipulation of virions through centrifugation and lysis, it is pertinent to acknowledge alternative approaches, such as immune complex dissociation utilized in previous studies for improving HIV antigen detection. Immune complex dissociation methods, as described in studies referenced [10, 11], and [12], have reported sensitivities ranging from 59 to 91.7%. That is to say, 59% reported by [10], 81% by [11], and 91.7% by [12] which are above the sensitivity found in this study. These methods typically involve the application of acid or heat treatment, often combined with signal amplification techniques, to enhance the detection of HIV antigens.

The observed differences in sensitivity between our study and those employing immune complex dissociation methods highlight the diversity of strategies employed to address the challenges associated with HIV antigen detection. While our approach yielded sensitivities lower than those reported in the aforementioned studies, it is essential to consider several factors that may contribute to this variation. These factors include differences in sample size, viral load levels, assay protocols, and patient populations.

Furthermore, it is worth noting that previous studies utilizing immune complex dissociation methods often involved complex and labor-intensive procedures, rendering them less suitable for resource-limited settings. In contrast, our modified technique offers a relatively simpler and more cost-effective approach, potentially making it more accessible in such settings.

### Limitations of the study

Direct comparison of results with the digital PCR was not done due to resource constraints and inaccessibility to the digital PCR equipment. Furthermore, the study did not conduct clinical validation involving PCR-based assays as reference standards as it was beyond the scope of the research project.

## Conclusion

This study clearly shows how the sample treatment procedures increase p24 antigen recovery in samples with very low viral loads that would otherwise be missed out without such treatments. The results of this study suggest that combining sample centrifugation with HIV-1 virion lysis could improve the detection of acute HIV-1 infection in blood for transfusion and even in pediatric patients especially in resource-limited African countries. Furthermore, the study has clearly shown that the performance of the 4th-generation Architect Ag/Ab combo assay for the detection of HIV-1 p24 Ag in acute HIV-1 infection may be comparable to that of PCR in the screening of blood for acute HIV infection in resource-limited settings. The findings might also greatly contribute to improvements in the monitoring and treatment management of HIV-1-infected persons in most resource-limited settings.

## Data Availability

Data associated with this study has been incorporated in this manuscript.

## Abbreviations

HIV: Human Immunodeficiency Virus
AIDS: Acquired Immunodeficiency Syndrome
Ag/Ab: Antigen/Antibody
WHO: World Health Organisation
S/CO: Signal - to - Cut off ratio
